# Inhibition of the PI3K/Akt/GSK3 Pathway Downstream of BCR/ABL, Jak2-V617F, or FLT3-ITD Downregulates DNA Damage-Induced Chk1 Activation as Well as G2/M Arrest and Prominently Enhances Induction of Apoptosis

**DOI:** 10.1371/journal.pone.0079478

**Published:** 2013-11-18

**Authors:** Tetsuya Kurosu, Toshikage Nagao, Nan Wu, Gaku Oshikawa, Osamu Miura

**Affiliations:** Department of Hematology, Graduate School of Medical and Dental Sciences, Tokyo Medical and Dental University, Tokyo, Japan; Innsbruck Medical University, Austria

## Abstract

Constitutively-activated tyrosine kinase mutants, such as BCR/ABL, FLT3-ITD, and Jak2-V617F, play important roles in pathogenesis of hematopoietic malignancies and in acquisition of therapy resistance. We previously found that hematopoietic cytokines enhance activation of the checkpoint kinase Chk1 in DNA-damaged hematopoietic cells by inactivating GSK3 through the PI3K/Akt signaling pathway to inhibit apoptosis. Here we examine the possibility that the kinase mutants may also protect DNA-damaged cells by enhancing Chk1 activation. In cells expressing BCR/ABL, FLT3-ITD, or Jak2-V617F, etoposide induced a sustained activation of Chk1, thus leading to the G2/M arrest of cells. Inhibition of these kinases by their inhibitors, imatinib, sorafenib, or JakI-1, significantly abbreviated Chk1 activation, and drastically enhanced apoptosis induced by etoposide. The PI3K inhibitor GD-0941 or the Akt inhibitor MK-2206 showed similar effects with imatinib on etoposide-treated BCR/ABL-expressing cells, including those expressing the imatinib-resistant T315I mutant, while expression of the constitutively activated Akt1-myr mutant conferred resistance to the combined treatment of etoposide and imatinib. GSK3 inhibitors, including LiCl and SB216763, restored the sustained Chk1 activation and mitigated apoptosis in cells treated with etoposide and the inhibitors for aberrant kinases, PI3K, or Akt. These observations raise a possilibity that the aberrant kinases BCR/ABL, FLT3-ITD, and Jak2-V617F may prevent apoptosis induced by DNA-damaging chemotherapeutics, at least partly through enhancement of the Chk1-mediated G2/M checkpoint activation, by inactivating GSK3 through the PI3K/Akt signaling pathway. These results shed light on the molecular mechanisms for chemoresistance of hematological malignancies and provide a rationale for the combined treatment with chemotherapy and the tyrosine kinase or PI3K/Akt pathway inhibitors against these diseases.

## Introduction

Constitutively-activated tyrosine kinase mutants play important roles in development and evolution of hematopoietic malignancies and are also implicated in acquisition of therapy resistance. The constitutively-activated fusion tyrosine kinase BCR/ABL is encoded by the fusion gene generated by a reciprocal t(9;22) (q34;q11.2) chromosomal translocation causing the Philadelphia chromosome (Ph), which is the molecular signature of chronic myeloid leukemia (CML) and is also observed in 30–40% of acute lymphoblastic leukemia (ALL) [1], [2]. BCR/ABL confers survival and proliferation advantages on hematopoietic cells by activating various intracellular signaling pathways, such as those involving Ras, Raf-1, MEK, Erk, phosphatidylinositol 3-kinase (PI3K), Akt, STAT5, and NFκB, which normally play roles in regulation of hematopoiesis by hematopoietic cytokine receptors that activate the Jak family tyrosine kinases, including Jak2 [1], [2]. An activated mutant of Jak2, Jak2-V617F, is found in more than 90% of polycythemia vera and about 50% of essential thrombocythemia or primary myelofibrosis and is implicated in pathogenesis and progression of these myeloproliferative neoplasms [3], [4]. Jak2-V617F also constitutively activates the various intracellular signaling pathways by coupling with hematopoietic cytokine receptors, such as those for erythropoietin (Epo) and thrombopoietin. The tyrosine kinase mutation most frequently found in acute myeloid leukemia (AML) is the internal tandem duplication (ITD) mutation of FLT3, a receptor tyrosine kinase that plays a critical role in regulation of hematopoietic progenitor cells [5], [6]. FLT3-ITD and FLT3 with an activating amino acid substitution in the tyrosine kinase domain, such as FLT3-D835Y, also constitutively activate the PI3K/Akt and MEK/Erk signaling pathways as well as STAT5 to stimulate proliferation and enhance survival of hematopoietic cells. Although controversial results have been reported for FLT3-D835Y, FLT3-ITD has been associated with therapy resistance and established as a poor prognostic factor for AML [6].

Various tyrosine kinase inhibitors that block the catalytic activity of these aberrant kinases have been in clinical use or under development in clinical studies [6]–[9]. The BCR/ABL inhibitor imatinib has demonstrated unprecedented efficacy for treatment of CML or Ph+ ALL [8]. However, the resistance to imatinib develops in significant portions of patients under treatment, especially in those with CML in advanced stages or with Ph+ ALL, mostly due to the emergence of mutations in the BCR/ABL kinase domain. These mutations include the clinically most important T315I mutation, which is also totally resistant to the second generation BCR/ABL inhibitors nilotinib and dasatinib. It has also been demonstrated that these inhibitors may not be able to eradicate leukemic stem cells to cure CML or Ph+ ALL [8], [9]. Inhibitors for Jak2-V617F and FLT3-ITD have not shown clinical efficacy as remarkable as the BCR/ABL inhibitors [6], [7]. Strategies to combine these tyrosine kinase inhibitors with chemotherapeutic agents to enhance therapeutic effects have been used successfully in some cases or under clinical trials [6], [9]. Molecular and cellular mechanisms for the efficacy of these combined strategies have remained to be elucidated.

Most chemotherapeutic agents induce DNA damages to activate apoptotic pathways in malignant cells [10]. However, DNA damages also elicit checkpoint responses that delay or arrest cell cycle progression until the cell has adequately repaired the DNA damage, thus mitigating chemotherapeutic effects [11], [12]. DNA damage checkpoints mainly induce G1/S arrest to prevent replication of damaged DNA or G2/M arrest to prevent segregation of damaged chromosomes during mitosis. While p53 plays a critical role in activation of G1/S checkpoint by inactivating the Cdk2 kinase through induction of the cdk inhibitor p21 expression, the G2/M arrest is dependent mainly on Chk1-mediated signaling pathway leading to inhibition of the Cyclin B1/Cdc2 activity [11]. Chk1, a serine/threonine kinase, is activated by phosphorylation on S317 and S345 by the DNA damage-activated ATR kinase and inhibits the Cdc25 phosphatases, thus inhibiting dephosphorylation of inhibitory phosphorylation of Cdc2 on Tyr15 and Thr14 to arrest the G2/M transition. Activated Chk1 is regulated through dephosphorylation by PP2A and other phosphatases and through ubiquitination and proteasomal degradation [11]. We previously showed that hematopoietic cytokines, such as IL-3 and Epo, enhance Chk1-mediated cell cycle checkpoint activation by the topoisomerase II inhibitor etoposide through inhibition of GSK3 by activating the PI3K/Akt pathway, thus inhibiting etoposide-induced apoptosis [13].

The present study reveals that the aberrant tyrosine kinases may endow resistance to chemotherapy on hematopoietic cells through similar mechanisms with the hematopoietic cytokines, because the inhibition of aberrant kinases or its downstream PI3K/Akt signaling pathway downregulated Chk1 activation as well as the G2/M cell cycle arrest and drastically enhanced apoptosis of hematopoietic cells treated with DNA-damaging chemotherapeutic agents. The present study would provide valuable data for development of the efficient combination therapies for hematopoietic malignancies associated with the aberrant tyrosine kinases including those with the kinase-inhibitor resistant mutations.

## Materials and Methods

### Cells and Reagents

A clone of murine IL-3-dependent BaF3 cells transfected with a BCR/ABL cDNA under the control of a tetracycline-inducible promoter was kindly provided by Dr. G. Daley [14]. Ton.B210 cells were cultured in 10% fetal calf serum (FCS)-containing RPMI 1640 medium supplemented either with 10% Wehi3B conditioned medium as the source of IL-3 or with 1 µg/ml doxycycline (DOX), which induces the expression of BCR/ABL. Ton.B210/T315I cells [15], which inducibly express BCR/ABL with the T315I mutation, and a human leukemic UT7 cells expressing the V617F mutant of Jak2, UT7/Jak2-V617F [16], as well as 32Dcl3 cells expressing BCR/ABL, Ton.32Dp210 [17], and the ITD or D835Y mutant of FLT3, Ton.32D/FLT3-ITD or Ton.32D/FLT3-D835Y [18], respectively, were described previously. The human CML cell line K562 and the FLT3-ITD-expressing AML cell line MV4-11 [19] were obtained from the Riken cell bank (Ibaraki, Japan) and from the American Type Culture Collection, respectively. PLAT-A [20], an amphotropic virus packaging cell line, was kindly provided by Dr. T. Kitamura, and maintained in DMEM medium supplemented with 10% FCS.

Imatinib was kindly provided by Novartis (Basel, Switzerland). Dasatinib and sorafenib were purchased from Toronto Research Chemicals Inc. (Toronto, Canada) and LTK Laboratories (St. Paul, MN), respectively. MK-2206 and GDC-0941 were purchased from Selleck (Houston, TX) and Chemdea (Ridgewood, NJ), respectively. Recombinant human Epo was kindly provided by Chugai Pharmaceutical Co. Ltd. (Tokyo, Japan). Murine IL-3 was purchased from Peprotech. DOX and propidium iodide (PI) were purchased from Sigma (St Louis, MO, USA). Etoposide, doxorubicin, and LiCl were purchased from Wako (Tokyo, Japan). The Jak2 inhibitor JakI-1, the Chk1 inhibitor SB218078, the GSK3 inhibitor SB216763, MG132, and nocodazole were purchased from Calbiochem (La Jolla, CA, USA). GSK3-inhibitor #5 (GSK3 I-#5) [21] was synthesized and kindly provided by Dr. H. Kagechika. DiOC6 was purchased from Invitrogen (Carlsbad, CA, USA).

Antibodies against Chk1 (SC8408), FLT3 (SC479), CrkL (SC319), c-Abl (SC131), and α-tubulin (SC5286) were purchased from Santa Cruz Biotechnology (Santa Cruz, CA, USA). An anti-phosphotyrosine monoclonal antibody (4G10, 05-321) and anti-phospho-S10-histone H3 (06-570) were purchased from Millipore (Billerica, MA). Antibodies against phospho-S345-Chk1 (CS2348), phospho-Y694-STAT5 (CS9359), phospho-Y245-c-Abl (CS2861), phospho-S21/9-GSK3α/ß (CS9331), GSK3ß (CS9315), and p53 (CS2524) were purchased from Cell Signaling Technology (Beverly, MA). Anti-Mdm2 (OP115) and anti-ß-actin were purchased from Calbiochem and Sigma, respectively. Anti-PARP (SA-250) was from Biomol (Plymouth Meeting, PA).

### Flow Cytometric Analyses for Cell Cycle and Apoptosis

For flow cytometric analyses of cell cycle and apoptosis, cells were resuspended in Krishan’s reagent (0.05 mg/ml PI, 0.1% Na citrate, 0.02 mg/ml ribonuclease A, 0.3% NP-40). After incubation for 30 min on ice, cells were analyzed by flow cytometry. For multivariate flow cytometric cell cycle analysis, cells were simultaneously stained for DNA content with PI and for histone H3 phosphorylated on S10 using specific primary antibodies and FITC-conjugated goat F(ab’)2 fragment anti-rabbit IgG (H+L) antibody (IM0833) from Beckman Coulter (Miami, FL, USA), essentially as described previously [13].

### Mitotic Index Assays

Cells were cultured for 16 h with 50 ng/ml nocodazole and etoposide or imatinib. Cytospin preparation prepared was fixed and stained with Wright’s stain solution. Mitotic cells were scored by randomly counting 200 cells by light microscopy to calculate the mitotic index (%).

### Immunoprecipitation and Immunoblotting

Cells were lysed and subjected to immunoprecipitation and immunoblotting as described previously [22]. The results shown are representative of experiments repeated at least three times.

### Expression Plasmids and Infection

Plasmids encoding wild type GSK3ß, kinase-inactive GSK3ß with the K85M and K69I mutations (GSK3ß-KI), and Akt-insensitive GSK3ß (GSK3ß-S9A) in the pCR3.1 vector were kindly provided by Dr. J. Sadoshima [23]. The inserts were cut out by *Nhe*I-*Not*I digestion and subcloned into the pMXs-IG retrovirus vector digested with *Stu*I and *Not*I to give pMXs-IG-GSK3ß, -GSK3ß-KI, and -GSK3ß-SA. A retroviral expression plasmid for constitutively activated Akt1, pRevTRE-Akt1-myr, has been described previously [16].

Transfection of these retroviral vectors into PLAT-A cells and infection of 32D/EpoR-Wt or Ton.32Dp210 cells were performed as described previously [18]. 32D/EpoR/pMXs-IG cells and the cells overexpressing GSK3ß-Wt, GSK3ß-KI, or GSK3ß-S9A were selected after infection by sorting GFP-expressing cells by flow cytometry. 32Dp210/Rev-Akt1-myr or the control 32Dp210/Rev cells were selected by puromycin.

## Results

### Inhibition of Jak2-V617F Prevents Chk1 Activation and G2/M Arrest in a GSK3-dependent Manner and Induces Apoptosis Drastically in Etoposide-treated Cells

**Figure 1:**
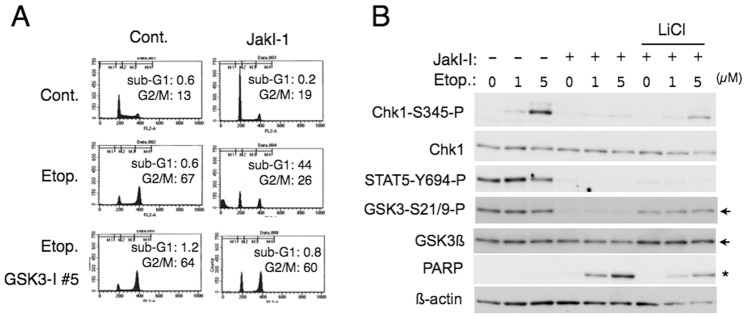
Inhibition of Jak2-V617F downregulates etoposide-induced Chk1 activation as well as G2/M arrest and elicits apoptosis in a GSK3-dependent manner. (**A**) UT7/Jak2-V617F cells were cultured for 16 h with 0.5 µM etoposide (Etop.), 0.2 µM JakI-1, or 1 µM GSK3-I #5, as indicated, in the absence of Epo. Cells were then analyzed for the cellular DNA content by flow cytometry. Percentages of apoptotic cells with sub-G1 DNA content (s-G1) and those of cells in the G2/M phase (G2/M) are indicated. (**B**) UT7-Jak2-V617F cells were pretreated for 60 min with 1 µM JakI-1 or 40 mM LiCl, as indicated, and subsequently treated with indicated concentrations of etoposide for 6 h. Cells were lysed and subjected to Western blot analysis with antibodies against indicated proteins. Positions of GSK3ß are indicated by arrows, while the caspase-cleaved fragment of PARP by an asterisk.

We previously reported that hematopoietic cytokines, such as IL-3 and Epo, enhance Chk1-mediated cell cycle checkpoint activation by etoposide through inhibition of GSK3 by activating the PI3K/Akt pathway, thus inhibiting etoposide-induced apoptosis [13]. Because these cytokines activate various intracellular signaling pathways mainly though coupling with the cytoplasmic tyrosine kinase Jak2, we fist analyzed UT7/Jak2-V617F [16], human leukemic UT7 cells expressing Jak2-V617F, to extend our previous observation. In contrast to parental UT7 cells, which undergo apoptosis without showing the G2/M arrest significantly after treatment with etoposide in the absence of Epo (Fig. S1A) [13], UT7/Jak2-V617F cells accumulated in the G2/M phase with only an insignificant portion of cells showing the sub-G1 DNA content, a hallmark of apoptotic cells, after etoposide treatment in the absence of Epo (Fig. 1A). However, the Jak2 inhibitor JakI-1 significantly inhibited the G2/M phase arrest and conspicuously induced apoptosis in etoposide-treated UT7/Jak2-V617F cells, whereas JakI-1 alone did not significantly induce apoptosis in these cells. Because signaling from the Epo receptor is mainly mediated through Jak2 activation, JakI-1 also significantly reduced the accumulation of parental UT7 cells treated with etoposide in the G2/M phase and modestly induced apoptosis (Fig. S1A). We next examined the effect of GSK3 inhibition and found that its specific inhibitor GSK3-I #5 prevented apoptosis induced by the combined treatment with etoposide and JakI-1 in UT7/Jak2-V617F cells and significantly restored the G2/M arrest (Fig. 1A), as expected from our previous observations [13].

We next examined the effect of JakI-1 on Chk1 activation by etoposide in these cells and found that it strongly inhibited the activation-specific phosphorylation of Chk1 on S345 after etoposide treatment, which was partially restored by cotreatment with the GSK3 inhibitor LiCl (Fig. 1B). JakI-1 also strongly inhibited the inhibitory phosphorylation of GSK3ß on S9 as well as activation-specific phosphorylation of the direct Jak2 substrate STAT5 on Y694, while LiCl restored the inhibitory phosphorylation of GSK3ß without showing any significant effect on STAT5. JakI-1 induced appearance of the caspase-cleaved fragment of PARP in etoposide-treated cells, which was significantly prevented by treatment with LiCl. As expected from our previous report [13], inhibition of Chk1 by its inhibitor SB218078 down regulated accumulation of etoposide-treated cells in the G2 or M phase and prominently enhanced apoptosis similarly with JakI-1 (Fig. S1B). JakI-1 as well as SB218078 was also confirmed to increase the expression level of histone H3 phosphorylated on S10, a well-established mitotic marker, in cells treated with etoposide and nocodazole, which traps cells in mitosis (Fig. S1C). These results suggest that, similarly with the hematopoietic cytokine stimulation leading to activation of Jak2, Jak2-V617F enhances etoposide-induced Chk1 activation possibly through inhibition of GSK3 to arrest cells at the G2/M phase and prevent apoptosis.

### Imatinib Attenuates the Chk1-mediated G2/M Cell Cycle Checkpoint Activation by Inhibiting BCR/ABL and Drastically Enhances Apoptosis in Cells Treated with Etoposide or Doxorubicin

**Figure 2:**
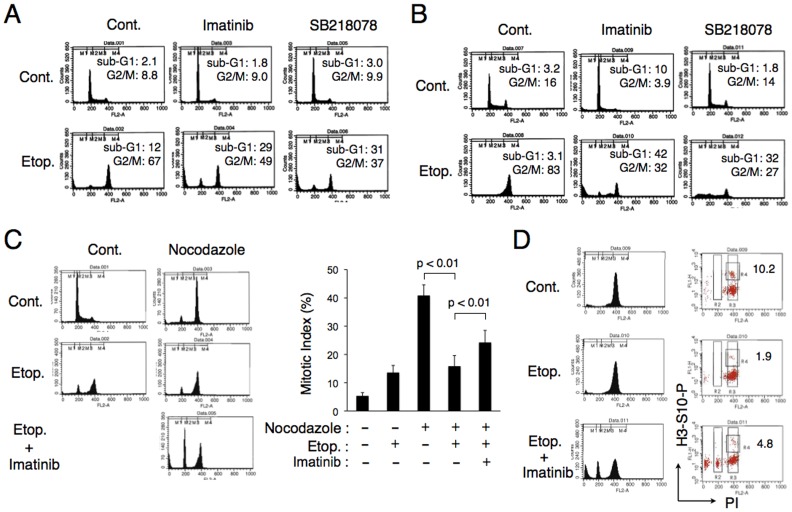
Imatinib inhibits the G2/M arrest and prominently induces apoptosis in BCR/ABL-expressing cells treated with etoposide. (**A**) 32Dp210 cells were cultured for 24 h with 0.5 µM etoposide (Etop.), 0.6 µM imatinib, or 1 µM SB218078, as indicated, and analyzed for the cellular DNA content by flow cytometry. Percentages of apoptotic cells with sub-G1 DNA content and those of cells in the G2/M phase are indicated. (**B**) K562 cells were cultured for 24 h with 1 µM etoposide, 1 µM imatinib, or 1 µM SB218078, as indicated, and analyzed. (**C**) 32Dp210 cells were cultured for 16 h with or without 50 ng/ml nocodazole in the presence of 1 µM etoposide and 1 µM imatinib, as indicated, and analyzed for the cellular DNA content by flow cytometry and for the mitotic index, as described under Materials and methods. Each data point represents the mean of three independent experiments, with error bars indicating standard deviations. The asterisk indicates a statistically significant difference determined by Student’s *t*-test (*p*<0.01). (**D**) 32Dp210 cells were cultured for 16 h with 1 µM etoposide and 1 µM imatinib, as indicated, in the presence of 50 ng/ml nocodazole. Cells were analyzed for the DNA content and histone H3 phosphorylated on S10 (H3-S10-P) by flow cytometry. Percentages of cells in G2/M that are positive for H3-S10-P are indicated.

To further extend our observation that normal or aberrant activation of proliferation signaling in hematopoietic cells enhances checkpoint activation by etoposide, we next examined cells expressing the aberrant fusion tyrosine kinase BCR/ABL. As shown in Fig. 2A, 32Dp210 cells transformed by BCR/ABL and growing in the absence of cytokines accumulated in the G2 or M phase after treatment with etoposide with only a modest portion of cells undergoing apoptosis. On the other hand, the Chk1 inhibitor SB218078 down regulated accumulation of etoposide-treated cells in the G2 or M phase and prominently enhanced apoptosis. The BCR/ABL inhibitor imatinib also significantly enhanced etoposide-induced apoptosis similarly with SB218078 in these cells. We also examined K562 cells and confirmed that imatinib inhibited accumulation of K562 cells in the G2 or M phase and drastically enhanced etoposide-induced apoptosis in this CML cell line in a similar manner with SB218078 (Fig. 2B).

To characterize more precisely the etoposide-induced accumulation of cells in the G2 or M phase and its inhibition by imatinib, we next treated 32Dp210 cells with nocodazole, a microtubule-disrupting agent, and measured mitotic indices with or without co-treatment with etoposide and imatinib. As shown in Fig. 2C, the percentage of cells arrested in mitosis by nocodazole treatment was conspicuously decreased by co-treatment with etoposide, although the flow cytometric analyses showed that the majority of cells were accumulated in the G2 or M phase, thus indicating that etoposide inhibited the G2 to M phase progression of these cells. However, the decrease in mitotic index by etoposide treatment was significantly inhibited by co-treatment with imatinib (Fig. 2C). Similarly, the Chk1 inhibitor SB218078 also induced a significant increase in mitotic index of cells treated with etoposide (Fig. S2A). We next examined the phosphorylation of histone H3 on S10, a common mitotic marker, in these cells by flow cytometry. As demonstrated in Fig. 2D, etoposide drastically reduced phospho-H3-S10, which was partly restored by co-treatment with imatinib and etoposide. SB218078 showed a similar effect with imatinib on phospho-H3-S10 as demonstrated by flow cytometry (Fig. S2B) and Western blot analyses (Fig. S2C). Taken together these results suggest that imatinib may inhibit the etoposide-induced and Chk1-mediated cell cycle arrest at the G2/M transition to induce apoptosis in BCR/ABL expressing cells.

**Figure 3:**
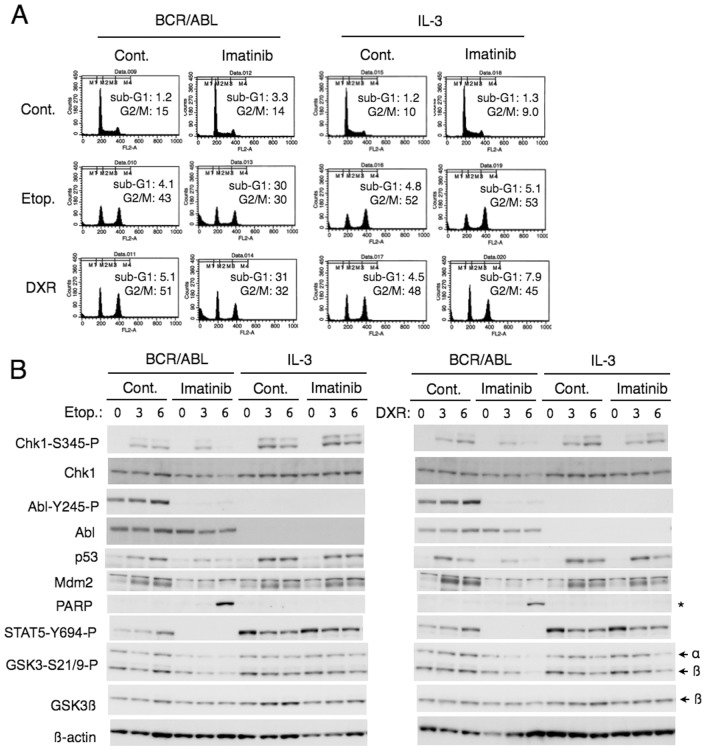
Imatinib downregulates etoposide- or doxorubicin-induced Chk1 activation and induces apoptosis specifically by inhibiting BCR/ABL. (**A**) Ton.B210 cells cultured with DOX to induce BCR/ABL expression (BCR/ABL) in the absence of IL-3 or cultured without DOX in the presence of IL-3 (IL-3) were left untreated as control (Cont.) or treated with 0.6 µM imatinib, 0.5 µM etoposide (Etop.), or 0.1 µM doxorubicin (DXR), as indicated for 12 h, and analyzed for the cellular DNA content. Percentages of apoptotic cells with sub-G1 DNA content and those of cells in the G2/M phase (G2/M) are indicated. (**B**) Ton.B210 cells expressing BCR/ABL (BCR/ABL) or cultured with IL-3 (IL-3) were left untreated as control (Cont.) or treated with 3 µM imatinib, as indicated, for 1 h. Cells were subsequently treated with 1 µM etoposide or 0.2 µM doxorubicin, as indicated, for indicated times. Cells were lysed and subjected to Western blot analysis with antibodies against indicated proteins. Positions of GSK3α and GSK3ß are indicated. A position of the caspase-cleaved fragment of PARP is indicated by an asterisk.

To exclude the possibility that imatinib may affect the checkpoint activation mechanisms through its inhibitory effects on tyrosine kinases other than BCR/ABL, such as c-Abl, we examined Ton.B210 cells, which inducibly express BCR/ABL when cultured with DOX. As shown in Fig. 3A, imatinib profoundly enhanced etoposide-induced apoptosis selectively in BCR/ABL-driven Ton.B210 cells but not in cells not expressing BCR/ABL and growing in the presence of IL-3. Very similar results were obtained when cells were treated with doxorubicin instead of etoposide (Fig. 3A). These results suggest that imatinib inhibits activation of the G2/M checkpoint and synergistically enhances apoptosis specifically through inhibition of BCR/ABL in cells treated not only with etoposide but also with doxorubicin.

We next examined the checkpoint activation mechanisms in Ton.B210 cells by Western blot analyses. As shown in Fig. 3B, activation of Chk1 by etoposide was shortened and not sustained in imatinib-treated BCR/ABL-driven cells, whereas imatinib did not show any significant effect on Chk1 activation by etoposide in the same clone of cells when they were not expressing BCR/ABL and cultured with IL-3. Imatinib also downregulated induction of p53, which is partly dependent on activation of Chk1 [11], and its target gene product Mdm2 specifically in BCR/ABL-driven cells. It was confirmed that imatinib induced appearance of the caspase-cleaved PARP fragment specifically in BCR/ABL-driven cells treated with etoposide. It was also confirmed that imatinib abrogated activation-specific phosphorylation of STAT5 as well as autophosphorylation of BCR/ABL specifically in cells driven by BCR/ABL. Imatinib also reduced inhibitory phosphorylation of GSK3 specifically in BCR/ABL-driven cells. Essentially the same results were obtained in Ton.B210 cells treated with doxorubicin (Fig. 3B). These results indicate that imatinib attenuates Chk1 activation involved in cell cycle checkpoint activated by etoposide or doxorubicin through its inhibitory effect on BCR/ABL.

### Sorafenib Downregulates Etoposide-induced Checkpoint Activation Mediated by Chk1 and Induces Apoptosis through Inhibition of FLT3-ITD

**Figure 4:**
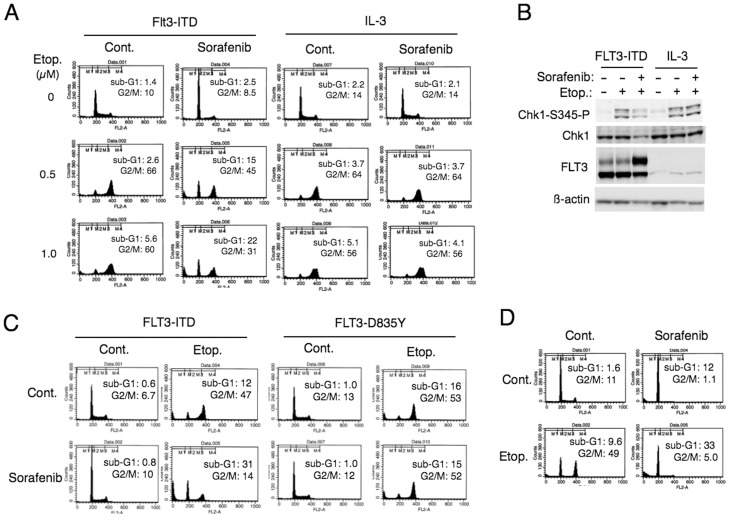
Sorafenib inhibits FLT3-ITD to attenuate Chk1 activation and induces apoptosis in cells treated with etoposide. (**A**) Ton.32D/FLT3-ITD cells cultured with DOX to induce FLT3-ITD (FLT3-ITD) in the absence of IL-3 or cultured without DOX in the presence of IL-3 (IL-3) were left untreated as control (Cont.) or treated with 0.5 µM sorafenib and indicated concentrations of etoposide (Etop.) for 16 h, and analyzed for the cellular DNA content. Percentages of apoptotic cells with sub-G1 DNA content and those of cells in the G2/M phase (G2/M) are indicated. (**B**) Ton.32D/FLT3-ITD cells expressing FLT3-ITD (FLT3-ITD) or cultured with IL-3 (IL-3) were left untreated or treated with 0.5 µM sorafenib and subsequently cultured with or without 0.5 µM etoposide, as indicated, for 10 h. Cells were lysed and subjected to Western blot analysis with antibodies against indicated proteins. (**C**) Ton.32D/Rev-FLT3-ITD cells (FLT3-ITD) or Ton.32D/Rev-FLT3-D835Y cells (FLT3-D835Y) were cultured with 50 nM sorafenib and 1 µM etoposide for 24 h and analyzed. (**D**) MV4-11 leukemic cells expressing FLT3-ITD werer cultured with 10 µM sorafenib and 0.2 µM etoposide, as indicated, for 24 h and analyzed.

To address the possibility that inhibition of other tyrosine kinase mutants in leukemic cells may also down regulate the Chk1 activation and sensitize cells to DNA damage-induced apoptosis, we examined Ton.32D/FLT3-ITD cells, which inducibly express FLT3-ITD and become IL3 independent when cultured with DOX. As shown in Fig. 4A, etoposide induced a pronounced accumulation of Ton.32D/FLT3-ITD cultured with DOX in the G2/M phase without inducing any significant apoptosis. However, a potent inhibitor of FLT3-ITD, sorafenib [6], [24], attenuated the etoposide-induced accumulation of cells in the G2/M phase and prominently induced apoptosis. On the other hand, sorafenib failed to sensitize Ton.32D/FLT3-ITD cells to etoposide-induced apoptosis when cells were cultured without DOX in the presence of IL-3, thus indicating that the sensitization may not be caused by non-specific inhibition of other kinases by this multi-kinase inhibitor. In these cells, sorafenib reduced the expression level of Chk1 and downregulated its activation-specific phosphorylation only when they were driven by FLT3-ITD (Fig. 4B). It was further demonstrated that sorafenib inhibited the etoposide-induced G2/M arrest and enhanced apoptosis specifically in cells driven by FLT3-ITD but not by FLT3-D835Y, which is resistant to sorafenib [24] (Fig. 4C). It was further confirmed that, similarly with sorafenib, the Chk1 inhibitor SB218078 downregulated the accumulation of etoposide-treated cell in G2/M phase and induced apoptosis (Fig. S3A). Moreover, sorafenib as well as SB218078 increased the expression level of histone H3 phosphorylated on S10 in cells treated with etoposide and nocodazole (Fig. S3B). These findings indicate that sorafenib downregulates Chk1-mediated checkpoint activation and induces apoptosis in etoposide-treated cells by specifically inhibiting FLT3-ITD. We also examined the human leukemic cell line MV4-11, which expresses FLT3-ITD [19]. As shown in Fig. 4D, sorafenib inhibited the G2/M phase accumulation and significantly enhanced apoptosis in MV4-11 cells treated with etoposide (Fig. 4D).

### Involvement of GSK3 Inhibition by the PI3K/Akt Pathway in Regulation of Etoposide-induced Chk1 Activation

**Figure 5:**
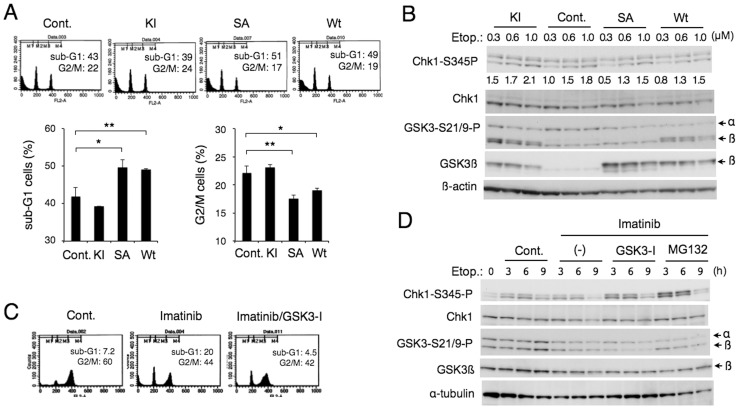
GSK3ß regulates etoposide-induced Chk1 activation and apoptosis in cytokine- or BCR/ABL-driven hematopoietic cells. (**A**) 32D/EpoR/pMXs-IG cells (Cont.) or the cells overexpressing the kinase inactive (KI) or S9A (SA) mutant as well as the wild-type (WT) GSK3ß, as indicated, were cultured for 16 h with 0.5 µM etoposide in the presence of 0.1 U/ml Epo and analyzed for the cellular DNA content. Percentages of apoptotic cells with sub-G1 DNA content and those of cells in the G2/M phase are plotted. Each data point represents the mean of three independent experiments, with error bars indicating standard deviations. The asterisks indicate statistically significant differences determined by Student’s *t*-test (*p<0.05, **p<0.01). (**B**) The cells indicated as in A were treated with indicated concentrations of etoposide for 7 h in the presence of 0.1 U/ml Epo and analyzed by Western blot analysis using indicated antibodies. Relative levels of Chk1-S345P, determined by densitometric analysis, are shown. (**C**) 32Dp210 cells were cultured for 16 h with 1 µM etoposide alone (Cont.) or also with 1 µM imatinib and 1 µM GSK3-I #5 (GSK3-I), as indicated, and analyzed for the cellular DNA content. (**D**) 32Dp210 cells were pretreated for 1 h with 1 µM imatinib, 1 µM GSK3-I #5, or 5 µM MG132, as indicated, or left untreated (Cont.). Cells were then treated with 1 µM etoposide for indicated times and analyzed by Western blot analysis.

We have shown that the inhibition of GSK3 is involved in enhancement of etoposide-induced Chk1 activation by Jak2-V617F (Fig. 1) as well as by cytokine receptor signaling [13] by using specific inhibitors. To confirm the involvement of GSK3, we overexpressed GSK3ß and its mutants in 32D/EpoR cells and examined their effect on etoposide-induced Chk1 activation. As shown in Fig. 5A and B, overexpression of wild-type GSK3ß or the S9A mutant, in which the inhibitory phosphorylation site is mutated, but not the inactive KI mutant, reduced the Chk1 activation and enhanced apoptosis in cells cultured with a low concentration of Epo and treated with etoposide.

We next examined the involvement of GSK3 in regulation of Chk1 activation in BCR/ABL-expressing cells. As shown in Fig. 5C, GSK3-I #5 prevented apoptosis induced in 32Dp210 cells by the combined treatment with etoposide and imatinib. Furthermore, the GSK3 inhibitor also prevented at least partly the inhibition of etoposide-induced Chk1 activation by imatinib in 32Dp210 cells (Fig. 5D). Because Chk1 is degraded through the ubiquitin/proteasome pathway [11], we also examined the effect of a proteasome inhibitor, MG132. MG132 suppressed the inhibition of Chk1 activation similarly with GSK3-I #5 and prevented a slight decline in its expression level by imatinib in etoposide-treated 32Dp210 cells (Fig. 5D). Thus, proteasomal degradation of Chk1 as well as GSK3 activation may be involved in negative regulation of etoposide-induced Chk1 by imatinib in these cells.

**Figure 6:**
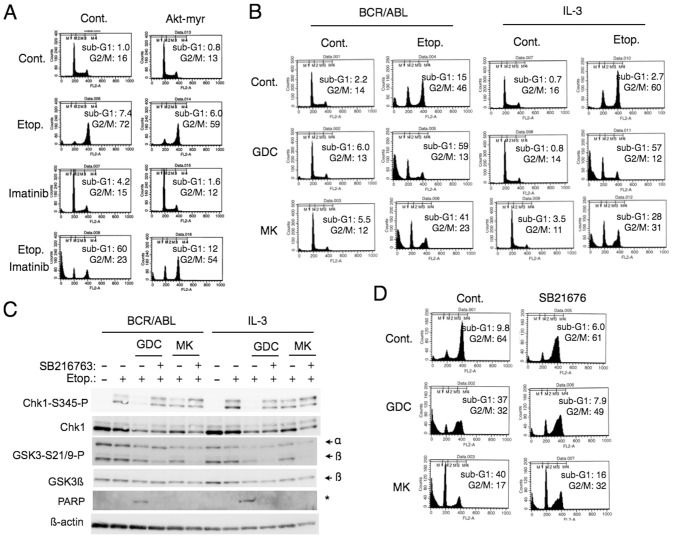
PI3K/Akt upstream of GSK3ß regulates etoposide-induced Chk1 activation and apoptosis in cytokine- or BCR/ABL-driven cells. (**A**) 32Dp210/Rev (Cont.) or 32Dp210/Rev-Akt1-myr (Akt-myr) cells were cultured with 0.5 µM etoposide (Etop.) or 2 µM imatinib, as indicated, for 24 h and analyzed for the cellular DNA content. (**B**) Ton.B210 cells cultured with DOX to induce BCR/ABL expression (BCR/ABL) in the absence of IL-3 or cultured without DOX in the presence of IL-3 (IL-3) were pretreated for 1 h with 1 µM GDC-0941 (GDC) or 5 µM MK-2206 (MK) or left untreated as control (Cont.). Cells were subsequently treated with 0.5 µM etoposide for 12 h or left untreated as control (Cont.), as indicated, and analyzed for the cellular DNA content. (**C**) Ton.B210 cells expressing BCR/ABL (BCR/ABL) or cultured with IL-3 (IL-3) were pretreated for 30 min with 2 µM GDC-0941, 3 µM MK-2206, or 20 µM SB216763, as indicated, or left untreated as control. Cells were subsequently treated with 1 µM etoposide for 8 h or left untreated, as indicated. Cells were lysed and subjected to Western blot analysis with antibodies against indicated proteins. A position of the caspase-cleaved fragment of PARP is indicated by an asterisk. (**D**) 32Dp210 cells were precultured for 1 h with 20 µM SB216763 or left untreated for control, as indicated. Cells were then treated for 24 h with 0.5 µM etoposide and 1 µM GDC-0941 or 5 µM MK-2206, as indicated, and analyzed.

To confirm the involvement of Akt upstream of GSK3 in regulation of Chk1 activation, we examined 32Dp210 cells engineered to express a constitutively activated Akt1 mutant, Akt1-myr. As shown in Fig. 6A, Akt1-myr prevented the synergistic induction of apoptosis by imatinib in etoposide-treated cells and retained the cells in the G2/M phase. We next confirmed the involvement of PI3K and Akt by using their specific inhibitors GDC-0941 and MK-2206, respectively, which are currently under clinical studies [25]–[29]. As shown in Fig. 6B, GDC-0941 or MK-2206 prevented the etoposide-induced G2/M arrest and drastically induced apoptosis in BaF3 cells driven by BCR/ABL or IL-3, while these inhibitors used alone did not conspicuously induced apoptosis in these cells. It was confirmed that GDC-0941 and, to a lesser extent, MK-2206 reduced the etoposide-induced Chk1 activation as well as inhibitory phosphorylation of GSK3 in these cells (Fig. 6C). It was further demonstrated that the GSK3 inhibitor SB216763 prevented the inhibition of Chk1 activation by GDC-0941 or MK-2206 (Fig. 6C). In agreement with these data, GDC-0941 increased the expression level of histone H3 phosphorylated on S10 similarly with imatinib in cells treated with etoposide, which was mostly prevented by SB216763 (Fig. S4). SB216763 also prevented appearance of the caspase-cleaved PARP fragment in cells treated with both etoposide and GCD-0941 (Fig. 6C). Flow cytometric analyses further demonstrated that SB216763 at least partly prevented the drastic enhancement of apoptosis induced by GDC-0941 or MK-2206 in etoposide-treated cells (Fig. 6D). These data agree with the hypothesis that BCR/ABL as well as hematopoietic cytokines inhibits the GSK3 activity through the PI3K/Akt pathway to prevent etoposide-induced apoptosis by maintaining Chk1-mediated G2/M checkpoint activation mechanisms.

### Sorafenib or the PI3K Inhibitor Downregulates Chk1 Activation and Profoundly Enhances Etoposide Sensitivity of Cells Expressing BCR/ABL with T315I

**Figure 7:**
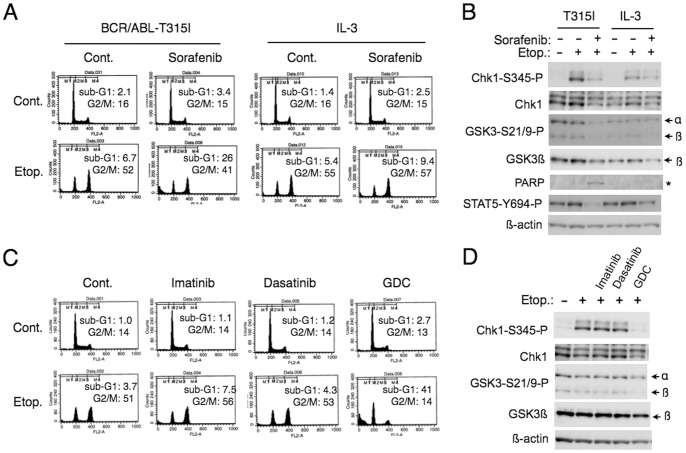
Sorafenib or GDC-0941 inhibits etoposide-induced Chk1 activation and enhances apoptosis in cells expressing T315I-mutated BCR/ABL. (**A**) Ton.B210/T315I cells cultured with DOX to induce BCR/ABL with T315I (BCR/ABL-T315I) in the absence of IL-3 or cultured without DOX in the presence of IL-3 (IL-3) were left untreated as control (Cont.) or treated with 5 µM sorafenib and 1 µM etoposide (Etop.), as indicated, for 16 h, and analyzed for the cellular DNA content. (**B**) Ton.B210/T315I cells expressing BCR/ABL with T315I (T315I) or cultured with IL-3 (IL-3) were left untreated or treated with 10 µM sorafenib, as indicated, for 1 h. Cells were subsequently cultured with or without 1 µM etoposide for 6 h and subjected to Western blot analysis. A position of the caspase-cleaved fragment of PARP is indicated by an asterisk. (**C**) Ton.B210/T315I cells cultured with DOX were left untreated as control (Cont.) or treated with 5 µM imatinib, 50 nM dasatinib, 1 µM GDC-0941 (GDC) in the presence or absence of 0.5 µM etoposide, as indicated, for 16 h, and analyzed for the cellular DNA content. (**D**) Ton.B210/T315I cells cultured with DOX were left untreated or treated with 5 µM imatinib, 50 nM dasatinib, 1 µM GDC-0941, as indicated, for 1 h. Cells were subsequently cultured with or without 1 µM etoposide for 6 h and subjected to Western blot analysis.

We finally examined cells expressing the T315I mutant of BCR/ABL, which is resistant not only to imatinib but also to dasatinib or nilotinib [8]. We first examined the effect of the multi-kinase inhibitor sorafenib, which we found to inhibit the kinase activity of BCR/ABL including the T315I mutant [17]. As shown in Fig. 7A and B, sorafenib much more significantly enhanced etoposide-induced apoptosis of BaF3 cells when they were driven by the T315I mutant as compared with when cultured with IL-3, which correlated with its inhibitory activity on Chk1 activation as well as on phosphorylation of the BCR/ABL substrate STAT5 and GSK3. These results suggest that sorafenib distinctively enhanced apoptosis in these cells by inhibiting the T315I mutant to attenuate the Chk1-mediated checkpoint activation.

We then examined the effect of GDC-0941 on cells expressing the T315I mutant. As shown in Fig. 7C, GDC-0941 but not imatinib or dasatinib inhibited the G2/M arrest induced by etoposide and prominently enhanced apoptosis in Ton.B210/T315I cells. It was confirmed that GDC-0941 but not imatinib or dasatinib inhibited the etoposide-induced Chk1 activation as well as phosphorylation of GSK3α/ß on S21/9 (Fig. 7D). Similar results were obtained with MK-2206 (data not shown). These date suggest that the major BCR/ABL mutant fully resistant to tyrosine kinase inhibitors could be sensitized to chemotherapeutics by inhibiting activation of the downstream PI3K/Akt pathway.

## Discussion

**Figure 8:**
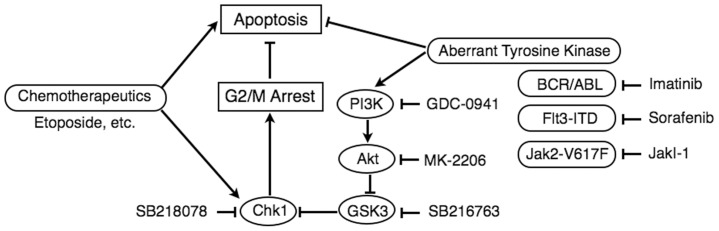
A schematic model for enhancement of apoptosis by inhibition of the aberrant tyrosine kinases in hematopoietic cells treated with chemotherapeutics. Chemotherapeutics induces Chk1-mediated G2/M cell cycle arrest to downregulate induction of apoptosis. Inhibition of BCR/ABL, FLT3-ITD, or Jak2-V617F by imatinib, sorafenib, of JakI-1, respectively, as well as inhibition of PI3K or Akt by GDC-0941 or MK-2206, respectively, drastically enhances apoptosis in hematopoietic cells treated with chemotherapeutics. The enhancing effect, prevented by inhibition of GSK3 by SB216763, may be at least partly due to the inhibition of PI3K/Akt pathway leading to activation of GSK3, which may prevent Chk1-mediated G2/M cell cycle arrest by chemotherapeutics. Inhibition of Chk1 by its inhibitor SB218078 also prominently enhances apoptosis induced by chemotherapeutics.

In the present study, we have demonstrated that inhibition of BCR/ABL, Jak2-V617F, and FLT3-ITD, which represent the most frequently found aberrantly activated tyrosine kinases in CML, BCR/ABL-negative myeloproliferative neoplasms, and AML, respectively, downregulated Chk1 activation as well as G2/M cell cycle arrest and drastically enhanced apoptosis induced by chemotherapeutic agents, etoposide and doxorubicin (Fig. 1–4). Inhibition of PI3K or Akt by specific inhibitors showed similar effects with that of aberrant kinases on activation of Chk1 and induction of apoptosis in cells treated with etoposide (Fig. 6). Furthermore, activation of GSK3, which is negatively regulated by the PI3K/Akt pathway, was shown to play a major role in regulation of the Chk1 and G2/M checkpoint activation mechanisms, because inhibition of GSK3 protected the etoposide-induced Chk1 activation, G2/M arrest, and viability of cells from inhibition of the aberrant tyrosine kinases or the PI3K/Akt pathway (Fig. 1, 5, 6). Together with our previous findings [13], these data raise the possibility that these leukemogenic tyrosine kinases as well as hematopoietic cytokines protect hematopoietic cells from DNA damage-induced apoptosis, at least partly, by enhancing Chk1-mediated G2/M arrest by inhibition of GSK3 through activation of the PI3K/Akt pathway (Fig. 8).

In contrast to our data, it has been reported that activation of the PI3K/Akt pathway inhibited Chk1 activation and overrode the G2/M arrest. Using the hematopoietic 32D cells expressing the Epo receptor, Quelle and his colleagues [30] showed that the G2/M phase accumulation of γ-irradiated cells cultured in the presence of Epo or IL-3 was observed only when the PI3K/Akt pathway was inactivated. Quelle and his colleagues [31] further reported that reactivation of the PI3K/Akt pathway in G2-arrested cells increased the Cdc2 activity and induced endoduplication and non-apoptotic cell death, which is also in apparent contradiction to our results. However, in accordance with our data, their data revealed that the PI3K inhibition actually reduced the sustained Chk1 activation up to 48 h after irradiation of these cells [31]. Because apoptosis was not significantly induced with or without inactivation of the PI3K/Akt pathway [30], the apparent discrepancy observed between their data and our previous as well as current data using essentially the same hematopoietic model system is most likely due to the differences in type and intensity of DNA-damaging stresses. In this regard, it is noteworthy that the conditions we utilized may be more relevant to chemotherapies against hematological malignancies, where the effects are mostly gained by induction of apoptosis [10].

It was also reported that Akt induced phosphorylation of Chk1 on S280 in non-hematopoietic cells, which prevented activation of Chk1 by inducing cytoplasmic translocation and ubiquitination [32], [33], although it has been later reported that Chk1 inhibition by Akt was not mediated by Chk1 phosphorylation on S280 [34], [35]. In contrast, it was recently reported that the Chk1 phosphorylation on S280 is mediated by p90Rsk in response to serum stimulation or UV irradiation in non-hematopoietic cells, such as HeLa or U2OS, and facilitates Chk1 activation by inducing its nuclear translocation [36]. More recently, Ray-David et al. [37] also reported that p90Rsk, but not Akt, which was activated by mitogens and oncogenes, phosphorylated Chk1 on S280 to downregulate its activation, which, however, reduced, but not enhanced, chemosensitivity of melanoma cells. Taken together, these results suggest that activation of Chk1 is regulated through a variety of different mechanisms to modulate apoptosis differently depending on types of cells and stimuli. In this regard, it should be noted that GDC-0941 or MK-2206, but not imatinib, enhanced Chk1 activation up to 6 h when BCR/ABL-expressing cells were treated with very low concentrations (0.1 and 0.2 µM) of etoposide (data not shown). Thus, the PI3K/Akt pathway may enhance Chk1 activation also in hematopoietic cells under some circumstances, although under the conditions more relevant to treatment of leukemia its inhibition downregulated Chk1 activation and G2/M checkpoint to enhance apoptosis. Thus, our current results may be relevant specifically to hematopoietic cells treated with chemotherapeutic agents, such as the topoisomerase II inhibitors and anthracyclines.

The molecular mechanisms involved in the enhancement of Chk1 activation by the aberrant tyrosine kinases by inactivating GSK3 through the PI3K/Akt pathway remain to be elucidated. In this regard, it should be noted that inhibition of the PI3K/Akt pathway as well as treatment with the tyrosine kinase inhibitors modestly decreased the expression level of Chk1. This could be explained by downregulation of the transcription of the Chk1 gene, because its promoter activity is enhanced by the E2F family of transcription factors in proliferating cells [38], which is regulated at least partly through the PI3K/Akt pathway [28]. It is also possible that inhibition of the PI3K/Akt pathway may facilitate degradation of Chk1. It has been reported that degradation of Chk1 through the proteasomal system is enhanced by DNA-damaging agents, including topoisomerase II inhibitors [11]. Consistently, we observed that the proteasomal inhibitor MG132 significantly enhanced Chk1 activation in etoposide-treated cells and prevented its inhibition by imatinib (Fig. 5D). Thus, it is possible that inhibition of the aberrant kinases may downregulate Chk1 activation in these cells at least partly through facilitation of its degradation through the proteasomal system. However, it is notable that inhibition of the aberrant kinases or the PI3K/Akt pathway more prominently reduced activation of Chk1 than its expression in these cells (Figs. 1B, 3B, 5D, 6C, 7B, D). Furthermore, inhibition of GSK3 prevented downregulation of activation-specific phosphorylation of Chk1 but not its expression in cells treated with etoposide and the PI3K/Akt inhibitors (Fig. 6C). Therefore, the aberrant kinases should regulate Chk1 activation in these cells mainly through modulation of the Chk1 activation mechanisms involving its phosphorylation. To elucidate the molecular mechanisms involved, further studies are needed to examine the possible effects of these kinases on various factors known to be involved in regulation of Chk1 activation, including ATR, claspin, and protein phosphatases, such as PP2A and Wip1 [11].

The present studies would provide valuable information for the development of new therapeutic strategies for refractory hematopoietic malignancies. Various Chk1 inhibitors have been developed and under clinical studies to enhance synergistically the effects of chemotherapeutics against various malignancies including AML [12], [39]. In this study, we demonstrated that the Chk1 inhibitor SB218078 and tyrosine kinase inhibitors very similarly downregulated etoposide-induced G2/M arrest and very strongly enhanced apoptosis in cells expressing aberrant kinases, including leukemic K562 cells (Fig. 2A, B, S1B, S3A). However, it should be emphasized that, as compared with the Chk1 inhibitors, the inhibitors of aberrant tyrosine kinases or the PI3K/Akt pathway should more efficiently enhance the effects of chemotherapeutics particularly on refractory hematopoietic malignancies because of their inhibitory effects also on various anti-apoptotic signaling pathways, including the most important PI3K/Akt pathway [28]. Further studies are definitely needed to elucidate the exact molecular mechanisms by which apoptosis is synergistically induced by chemotherapeutics and inhibitors for the aberrant tyrosine kinases or the PI3K/Akt pathway.

Various inhibitors of PI3K or Akt currently under clinical studies [26], [28], [29] should be useful for chemosensitization of leukemias with tyrosine kinase domain mutations resistant to various inhibitors at least partly though downregulation of Chk1 activation. We previously revealed that sorafenib inhibited the tyrosine kinase activity of BCR/ABL including the T315I mutant [17], which is fully resistant to various tyrosine kinase inhibitors, such as imatinib and dasatinib. However, cells expressing the T315I mutant were also relatively resistant to sorafenib at 5 µM, which corresponds to the steady-state plasma concentration attainable in most patients continuously taking the standard dose (400 mg bid) of sorafenib [40]. In the present study, we demonstrated that sorafenib at 5 µM profoundly enhanced etoposide-induced apoptosis in BCR/ABL-T315I-transformed cells but not in IL-3-driven cells (Fig. 7A, B). Thus, sorafenib combined with chemotherapeutics may prove to be an effective therapeutic strategy against the refractory T315I-positive leukemias without significantly aggravating myelosuppression. Similarly, sorafenib or other FLT3 inhibitors under clinical studies may synergistically enhance the therapeutic efficacies of chemotherapeutics by downregulating Chk1-mediated G2/M arrest specifically in refractory AML cases with FLT3-ITD. These possibilities warrant clinical trials in a near future.

## Supporting Information

Figure S1 **Effects of inhibitors for Jak2 or Chk1 on etoposide-treated UT7 or UT7/Jak2-V617 cells.** **(A)** UT7 cells were cultured in the presence or absence of 1 U/ml Epo for 16 h with 0.5 µM etoposide (Etop.) and 0.2 µM JakI-1, as indicated. Cells were then analyzed for the cellular DNA content by flow cytometry. Percentages of apoptotic cells with sub-G1 DNA content (s-G1) and those of cells in the G2/M phase (G2/M) are indicated. **(B)** UT7/Jak2-V617F cells were cultured for 16 h with 0.5 µM etoposide (Etop.), 0.2 µM JakI-1, or 1 µM SB218078, as indicated, in the absence of Epo. Cells were then analyzed for the cellular DNA content by flow cytometry. **(C)** UT7/Jak2-V617F cells were cultured with 0.2 µM JakI-1 or 1 µM SB218078, as indicated, in the presence of 1 µM etoposide and 50 ng/ml nocodazole for 16 h. Cells were lysed and subjected to Western blot analysis with antibodies against histone H3 phosphorylated on S10 (H3-S10-P) and ß-actin, as indicated.
(TIF)

Figure S2 **The Chk1 inhibitor SB218078 inhibits the G2/M arrest similarly with imatinib in BCR/ABL-expressing cells treated with etoposide. (A)** 32Dp210 cells were cultured for 16 h with or without 1 µM SB218078, as indicated, in the presence of 50 ng/ml nocodazole and 1 µM etoposide. Cells were analyzed for the cellular DNA content by flow cytometry and for the mitotic index, as described under Materials and methods. Each data point represents the mean of three independent experiments, with error bars indicating standard deviations. The asterisk indicates a statistically significant difference determined by Student’s *t*-test (*p*<0.01). **(B)** 32Dp210 cells were cultured for 16 h with 1 µM etoposide and 0.5 µM SB218078, as indicated, in the presence of 50 ng/ml nocodazole. Cells were analyzed for the DNA content and histone H3 phosphorylated on S10 (H3-S10-P) by flow cytometry. Percentages of cells in G2/M that are positive for H3-S10-P are indicated. **(C)** 32Dp210 cells were cultured with 0.6 µM imatinib or 1 µM SB218078, as indicated, in the presence of 1 µM etoposide and 50 ng/ml nocodazole for 16 h. Cells were lysed and subjected to Western blot analysis with antibodies against indicated proteins.
(TIF)

Figure S3 **SB218078 inhibits the G2/M arrest and induces apoptosis similarly with sorafenib in FLT3-ITD-expressing cells treated with etoposide. (A)** Ton.32D/FLT3-ITD cells driven by FLT3-ITD were left untreated as control (Cont.) or treated with 0.5 µM sorafenib or 0.2 µM SB218078 in the presence or absence of 1 µM etoposide (Etop.), as indicated. Cells were then analyzed for the cellular DNA content by flow cytometry. **(B)** Ton.32D/FLT3-ITD cells were cultured with 0.5 µM sorafenib or 1 µM SB218078, as indicated, in the presence of 1 µM etoposide and 50 ng/ml nocodazole for 16 h. Cells were lysed and subjected to Western blot analysis with antibodies against indicated proteins.
(TIF)

Figure S4 **The PI3K inhibitor GDC-0941 induces expression of the mitotic marker histone H3 phosphorylated on S10 similarly with imatinib in BCR/ABL-expressing cells treated with etoposide.** 32Dp210 cells were cultured with 0.6 µM imatinib **(A)**, 1 µM GDC-0941 **(B)**, or 1 µM SB216763, as indicated, in the presence of 1 µM etoposideand for 16 h. Cells were lyzed and subjected to Western blot analysis with antibodies against indicated proteins.
(TIF)
