# Supplementary figures and images for: Inhibition of the PI3K/Akt/GSK3 Pathway Downstream of BCR/ABL, Jak2-V617F, or FLT3-ITD Downregulates DNA Damage-Induced Chk1 Activation as Well as G2/M Arrest and Prominently Enhances Induction of Apoptosis
Source: PLoS One. 2013 Nov 18;8(11):e79478. doi: 10.1371/journal.pone.0079478 (PMC3832535; doi:10.1371/journal.pone.0079478)

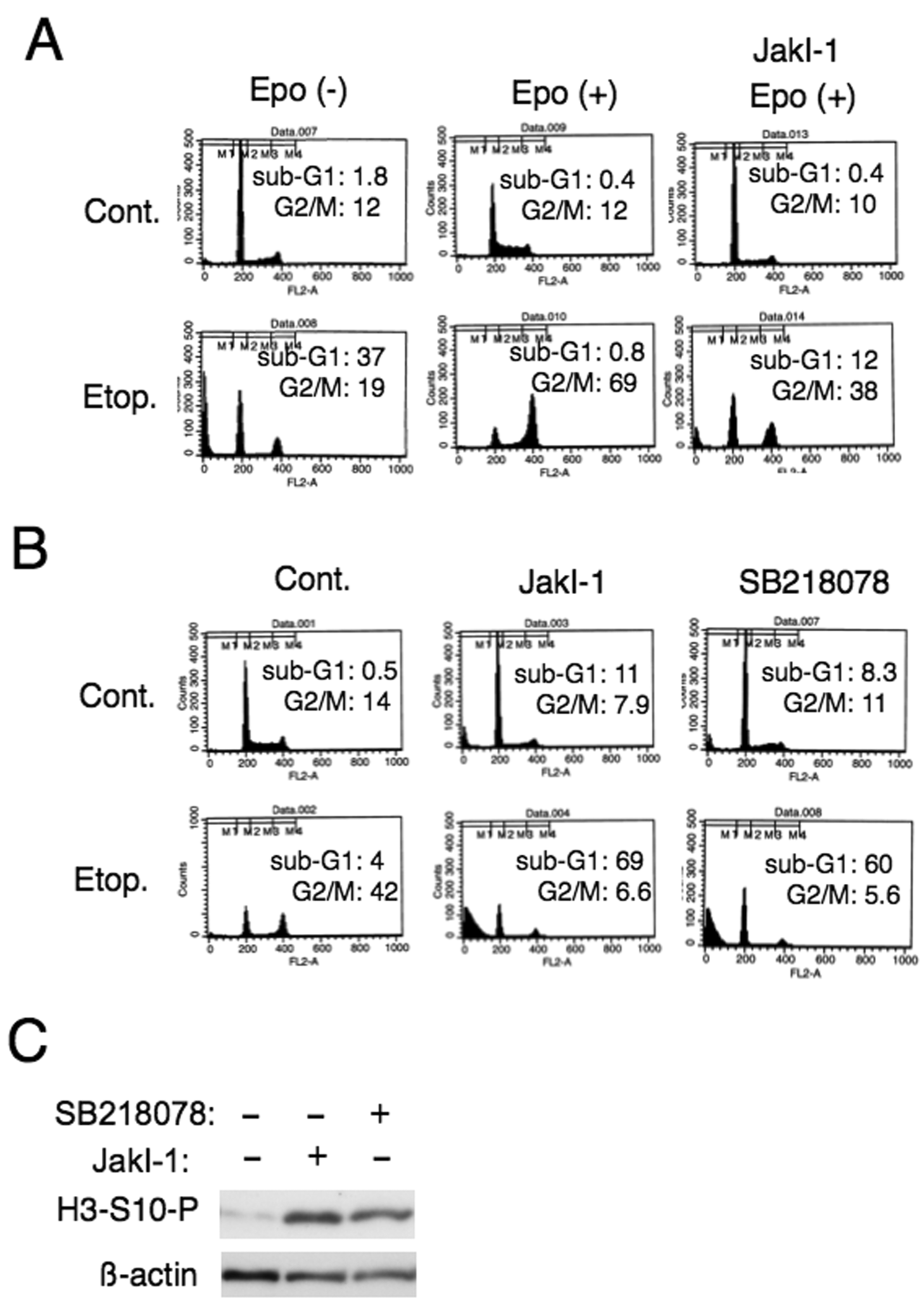

Supplement: Figure S1 — Effects of inhibitors for Jak2 or Chk1 on etoposide-treated UT7 or UT7/Jak2-V617 cells. (A) UT7 cells were cultured in the presence or absence of 1 U/ml Epo for 16 h with 0.5 µM etoposide (Etop.) and 0.2 µM JakI-1, as indicated. Cells were then analyzed for the cellular DNA content by flow cytometry. Percentages of apoptotic cells with sub-G1 DNA content (s-G1) and those of cells in the G2/M phase (G2/M) are indicated. (B) UT7/Jak2-V617F cells were cultured for 16 h with 0.5 µM etoposide (Etop.), 0.2 µM JakI-1, or 1 µM SB218078, as indicated, in the absence of Epo. Cells were then analyzed for the cellular DNA content by flow cytometry. (C) UT7/Jak2-V617F cells were cultured with 0.2 µM JakI-1 or 1 µM SB218078, as indicated, in the presence of 1 µM etoposide and 50 ng/ml nocodazole for 16 h. Cells were lysed and subjected to Western blot analysis with antibodies against histone H3 phosphorylated on S10 (H3-S10-P) and ß-actin, as indicated. (TIF) [file pone.0079478.s001.tif]

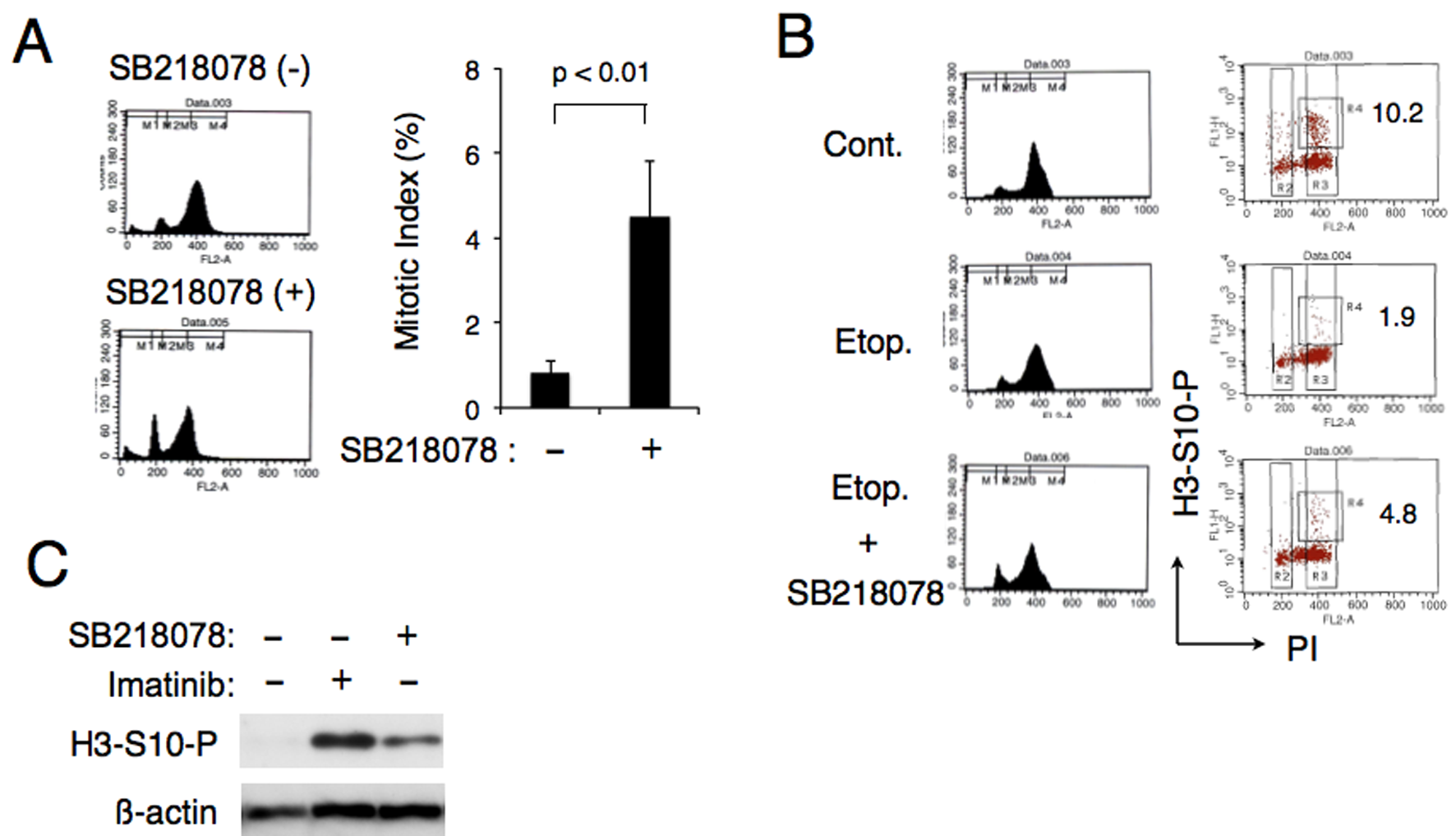

Supplement: Figure S2 — The Chk1 inhibitor SB218078 inhibits the G2/M arrest similarly with imatinib in BCR/ABL-expressing cells treated with etoposide. (A) 32Dp210 cells were cultured for 16 h with or without 1 µM SB218078, as indicated, in the presence of 50 ng/ml nocodazole and 1 µM etoposide. Cells were analyzed for the cellular DNA content by flow cytometry and for the mitotic index, as described under Materials and methods. Each data point represents the mean of three independent experiments, with error bars indicating standard deviations. The asterisk indicates a statistically significant difference determined by Student’s t-test (p<0.01). (B) 32Dp210 cells were cultured for 16 h with 1 µM etoposide and 0.5 µM SB218078, as indicated, in the presence of 50 ng/ml nocodazole. Cells were analyzed for the DNA content and histone H3 phosphorylated on S10 (H3-S10-P) by flow cytometry. Percentages of cells in G2/M that are positive for H3-S10-P are indicated. (C) 32Dp210 cells were cultured with 0.6 µM imatinib or 1 µM SB218078, as indicated, in the presence of 1 µM etoposide and 50 ng/ml nocodazole for 16 h. Cells were lysed and subjected to Western blot analysis with antibodies against indicated proteins. (TIF) [file pone.0079478.s002.tif]

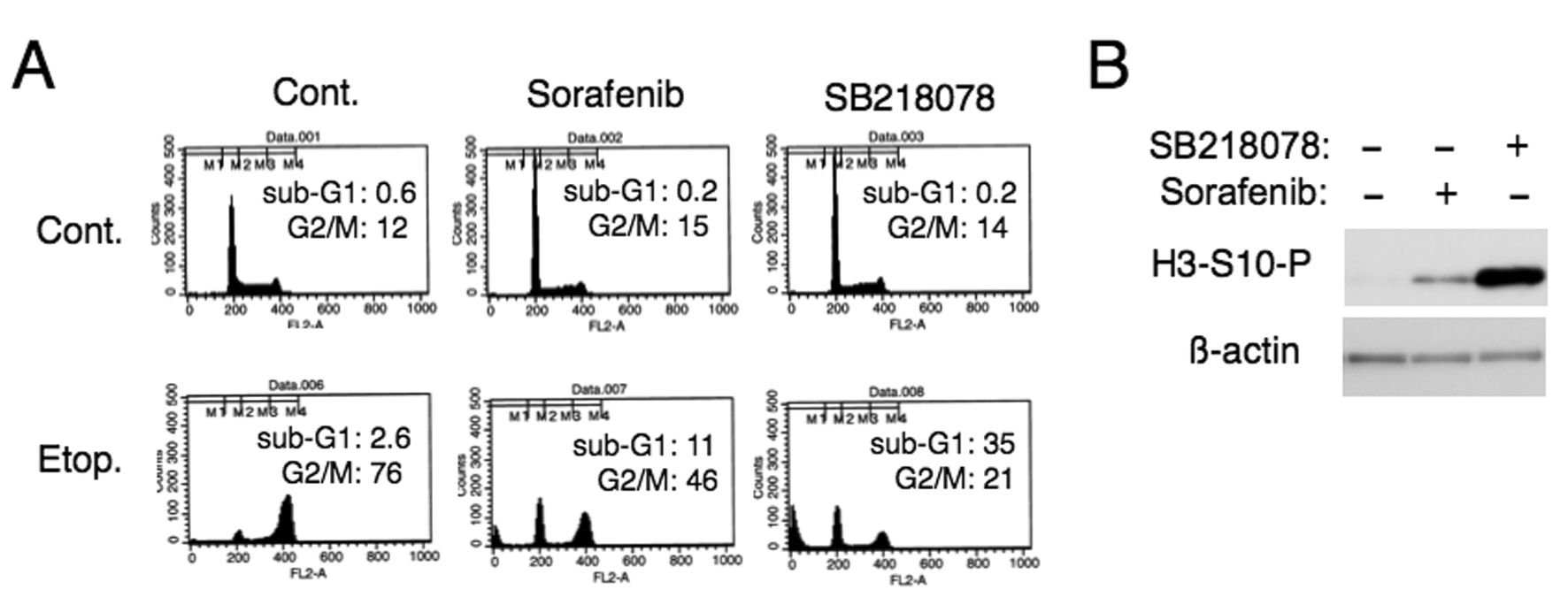

Supplement: Figure S3 — SB218078 inhibits the G2/M arrest and induces apoptosis similarly with sorafenib in FLT3-ITD-expressing cells treated with etoposide. (A) Ton.32D/FLT3-ITD cells driven by FLT3-ITD were left untreated as control (Cont.) or treated with 0.5 µM sorafenib or 0.2 µM SB218078 in the presence or absence of 1 µM etoposide (Etop.), as indicated. Cells were then analyzed for the cellular DNA content by flow cytometry. (B) Ton.32D/FLT3-ITD cells were cultured with 0.5 µM sorafenib or 1 µM SB218078, as indicated, in the presence of 1 µM etoposide and 50 ng/ml nocodazole for 16 h. Cells were lysed and subjected to Western blot analysis with antibodies against indicated proteins. (TIF) [file pone.0079478.s003.tif]

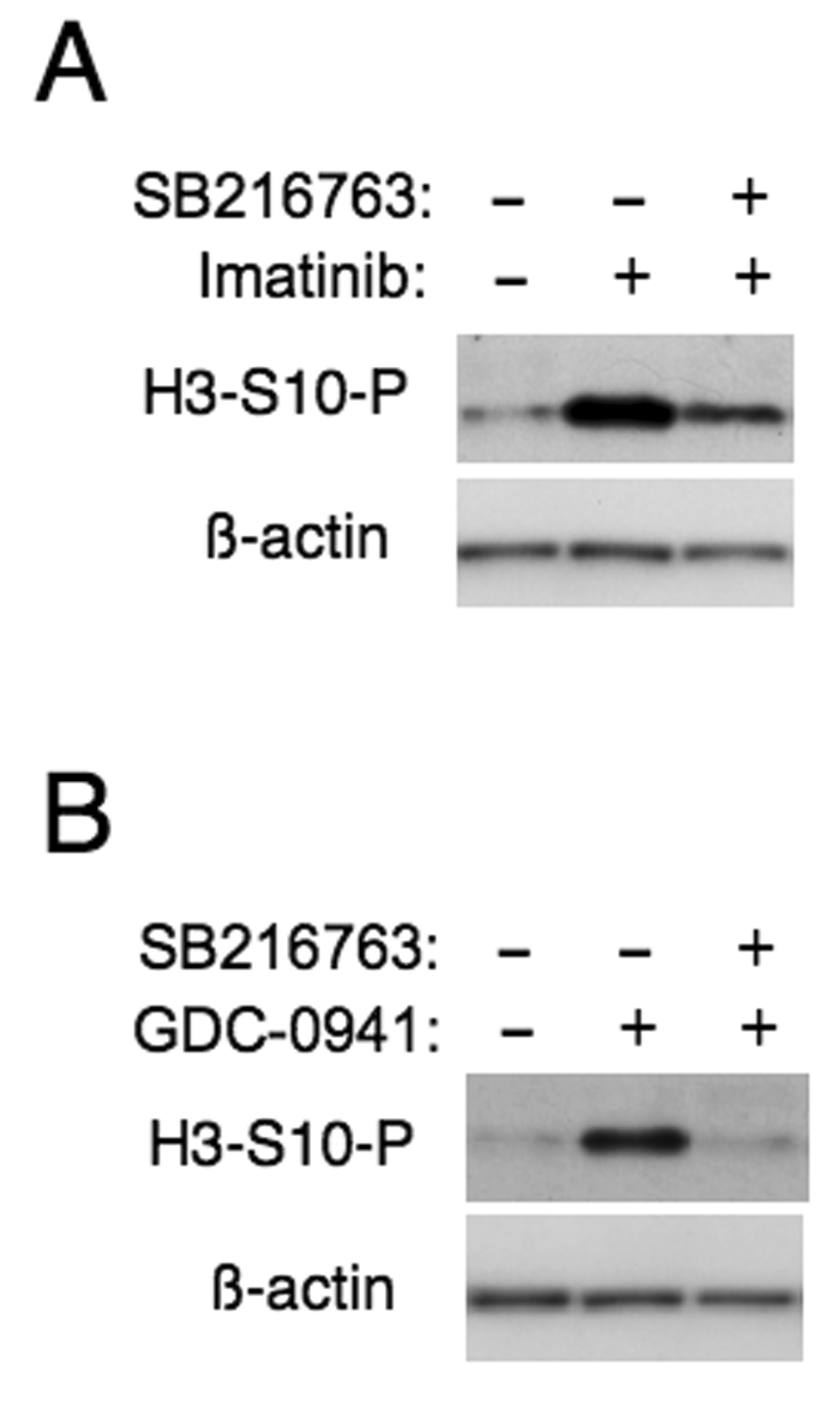

Supplement: Figure S4 — The PI3K inhibitor GDC-0941 induces expression of the mitotic marker histone H3 phosphorylated on S10 similarly with imatinib in BCR/ABL-expressing cells treated with etoposide. 32Dp210 cells were cultured with 0.6 µM imatinib (A), 1 µM GDC-0941 (B), or 1 µM SB216763, as indicated, in the presence of 1 µM etoposideand for 16 h. Cells were lyzed and subjected to Western blot analysis with antibodies against indicated proteins. (TIF) [file pone.0079478.s004.tif]
